# Supplementary material for: Causal Relationship between Aging and Anorexia Nervosa: A White-Matter-Microstructure-Mediated Mendelian Randomization Analysis
Source: Biomedicines. 2024 Aug 16;12(8):1874. doi: 10.3390/biomedicines12081874 (PMC11351342; doi:10.3390/biomedicines12081874)
Supplement: Supplementary file 1 [file biomedicines-12-01874-s001.zip › Supplementary Figure/Figure S4/Singe SNP plot of MR analysis for TL to ubm-b-1652.pdf]

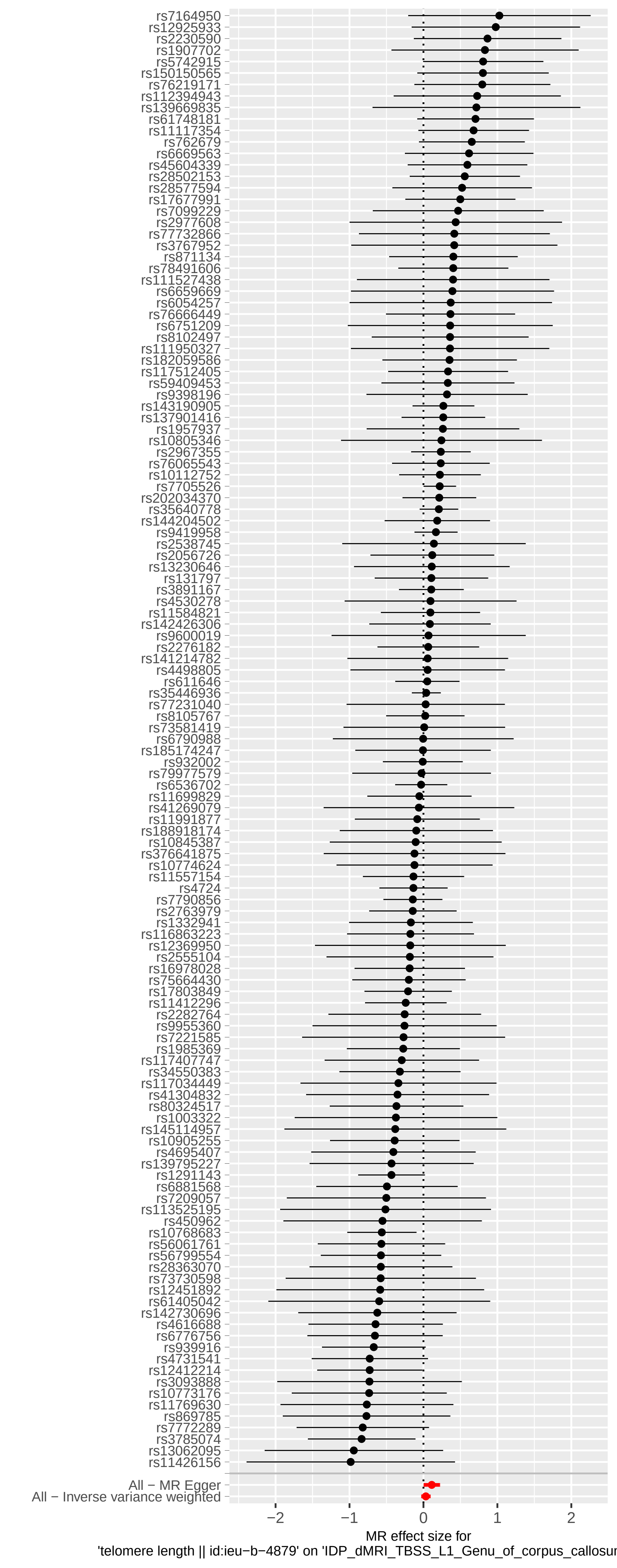
rs7164950
rs12925933
rs2230590
rs1907702
rs5742915
rs150150565
rs76219171
rs112394943
rs139669835
rs61748181
rs11117354
rs762679
rs6669563
rs45604339
rs28502153
rs28577594
rs17677991
rs7099229
rs2977608
rs77732866
rs3767952
rs871134
rs78491606
rs111527438
rs6659669
rs6054257
rs76666449
rs6751209
rs8102497
rs111950327
rs182059586
rs117512405
rs59409453
rs9398196
rs143190905
rs137901416
rs1957937
rs10805346
rs2967355
rs76065543
rs10112752
rs7705526
rs202034370
rs35640778
rs144204502
rs9419958
rs2538745
rs2056726
rs13230646
rs131797
rs3891167
rs4530278
rs11584821
rs142426306
rs9600019
rs2276182
rs141214782
rs4498805
rs611646
rs35446936
rs77231040
rs8105767
rs73581419
rs6790988
rs185174247
rs932002
rs79977579
rs6536702
rs11699829
rs41269079
rs11991877
rs188918174
rs10845387
rs376641875
rs10774624
rs11557154
rs4724
rs7790856
rs2763979
rs1332941
rs116863223
rs12369950
rs2555104
rs16978028
rs75664430
rs17803849
rs11412296
rs2282764
rs9955360
rs7221585
rs1985369
rs117407747
rs34550383
rs117034449
rs41304832
rs80324517
rs1003322
rs145114957
rs10905255
rs4695407
rs139795227
rs1291143
rs6881568
rs7209057
rs113525195
rs450962
rs10768683
rs56061761
rs56799554
rs28363070
rs73730598
rs12451892
rs61405042
rs142730696
rs4616688
rs6776756
rs939916
rs4731541
rs12412214
rs3093888
rs10773176
rs11769630
rs869785
rs7772289
rs3785074
rs13062095
rs11426156
All − MR Egger
All − Inverse variance weighted
−2
−1
0
1
2
MR effect size for
'telomere length || id:ieu−b−4879' on 'IDP_dMRI_TBSS_L1_Genu_of_corpus_callosur
